# Assessing Intervertebral Disk Tissue Mechanics Using Dual-Actuator Multifrequency Magnetic Resonance Elastography: Case Reports

**DOI:** 10.1155/carm/5383659

**Published:** 2025-04-13

**Authors:** Johannes Castelein, Tue Secher Jensen, Ronald Borra, Karen Kettless, Lau Brix, Greg Kawchuk

**Affiliations:** ^1^Department of Radiology, University Medical Center Groningen, Groningen, The Netherlands; ^2^2nd Department of Radiology, University Medical Center Gdańsk, Gdańsk, Poland; ^3^Department of Radiology, Silkeborg Regional Hospital, Silkeborg, Denmark; ^4^Department of Research and Collaboration, Siemens Healthcare A/S, Ballerup, Denmark; ^5^Department of Clinical Medicine, Aarhus University, Aarhus, Denmark; ^6^Department of Physical Therapy, University of Alberta, Edmonton, Canada

## Abstract

**Background:** Degenerative disk disease (DDD) is a progressive condition that occurs when the intervertebral discs (IVDs), which act as shock absorbers between the vertebrae, degenerate or wear out. Due to this degeneration process, the mechanical properties of the IVD, providing flexibility between adjacent vertebrae, can change. Thus, assessing these mechanical properties may improve diagnosis and treatment guidance for DDD. In this article, we tested in vivo multifrequency magnetic resonance elastography (MMRE) of the human IVD in identifying progressively DDD in three asymptomatic male volunteers aged 32, 50, and 60 years.

**Methods:** MMRE of the lumbar spine was acquired using a dual-actuator setup and operated at four frequencies from 60 to 90 Hz. MMRE data were postprocessed using multifrequency wave-number recovery (k-MDEV) inversion algorithm. The resulting shear wave speed (SWS) values were used as a surrogate parameter of tissue stiffness and then compared to Pfirrmann grading (Pf) of disc degeneration (1–5) performed by an experienced MRI spine researcher.

**Results:** Morphological Pf demonstrated an inverse relationship between increasing IVD stiffness and progressive IVD degeneration by a Spearman's rank correlation coefficient of *ρ* = −0.792, *p* < 0.001.

**Conclusion:** MMRE allows measurement of in vivo mechanical properties of IVDs and may provide additional information in disc degeneration beyond standard morphological changes. Prior to the clinical use of this technique, future studies should be conducted to evaluate the reproducibility and repeatability of spinal MMRE in the spine, and particularly its potential confounders.

## 1. Introduction

The intervertebral discs (IVDs) within the spinal column are characterized by a central region, known as the nucleus pulposus (NP), which is rich in proteoglycans and water, encapsulated by the annulus fibrosus (AF), a fibrous layer composed of collagen fibrils arranged in alternating lamellae [1]. This complex architecture enables the disc to support loads up to five times the body's weight during normal activities, while still facilitating spinal movement [2]. However, with advancing age [3] or following an injury [4, 5], the degeneration of the IVDs and adjacent spinal tissues leads to degenerative disk disease (DDD) which may cause pain and discomfort in the back and extremities [6, 7].

The Pfirrmann grading system is a widely recognized for its ability to classify DDD due to its reliability and feasibility [8]. In accordance with standard routine T2 turbo spin echo (TSE) weighted magnetic resonance imaging (MRI), the brightness distinction between annulus and nucleus is used to determine the Pfirrmann grade (1–5). Criteria for Pfirrmann grade are described in Yu et al. [9]. While the Pfirrmann score is commonly utilized, it is important to note that it is based on the morphological MRI appearance of the IVD and does not necessarily correlate with the severity of symptoms or the likelihood of future herniation events. The function of the IVD is more closely tied to its mechanical properties, which include the absorption of mechanical energy, providing flexibility between adjacent vertebrae [10]. As discs degenerate nutrient and water penetration are disrupted, leading to tissue remodeling, diminished vascularity, and altered mechanical properties [11]. Therefore, understanding alterations in material properties such as stiffness and viscosity may provide insights into the pathophysiology of disc degeneration and have the potential to improve diagnosis and guide treatment strategies.

Toward this, MR elastography (MRE) enables the quantification of tissue stiffness by utilizing low-frequency, noninvasive vibration to the region of interest. MRE was developed originally to stage liver fibrosis [12] and later applied to other tissues such as the brain [13] and kidneys [14]. Unfortunately, conventional single-frequency MRE may be limited for use in routine spinal diagnostics due to limitations such as displacement within deep spinal tissues. Although the lowest frequency in a multifrequency approach would likely penetrate deepest into spinal tissues, the combination of multiple frequencies can provide a more robust assessment of tissue properties, and the ability to analyze frequency-dependent behavior [10, 15]. Therefore, we describe here a proof-of-concept using a newly developed multifrequency MRE (MMRE) system. Employing a dual-actuator design, the system enables multifrequency, large-magnitude displacements within deep spinal tissues. In combination with a multifrequency wave-number recovery (k-MDEV) inversion algorithm [16], our proposed setup enables the acquisition of elasticity within low-SNR regions such as IVD or the spinal cord. The present case report demonstrates how an optimized MMRE setup can identify progressively increasing DDD.

## 2. Case Presentation

**Figure 1:**
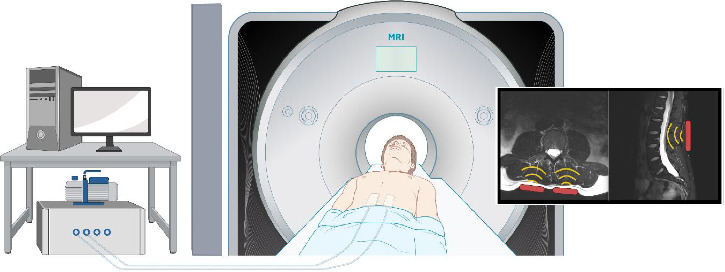
Experimental setup of MMRE of the human IVD. Two pneumatic actuators were placed bilaterally on the paravertebral muscles centered at the third lumbar level inducing compression waves (yellow) to the tissue.

Informed consent was obtained for three asymptomatic adult males aged 32, 50, and 60 years (University of Alberta Human Research Ethics Board Pro00119359). All data were processed and stored confidentially, and personal data were handled in accordance with the Personal Information Protection Act (PIPA Alberta). Stored data werepseudo-anonymoused in a separate approved and secure database using an anonymous trial number as subject identification. Participants were scanned in the Peter S. Allen MR Research Centre. Standard Sagittal T2 Dixon MRI images were obtained from a clinical 3T MRI system (Magnetom Prisma, Siemens Healthineers, Erlangen, Germany) using an 18Ch-body coil. The 3-D wave fields were acquired by echo-planar imaging (EPI) with flow-compensated motion-encoding gradients. Eight wave-phase offsets were recorded for each of the three motion directions. Four vibration frequencies were applied sequentially at 60, 70, 80, and 90 Hz using two pneumatic actuators placed bilaterally on the paravertebral muscles centered at the third lumbar level as shown in Figure 1. Postprocessing of the MMRE data was performed using a publicly available, k-MDEV [16] inversion algorithm (https://bioqic-apps.charite.de) resulting in shear wave speed (SWS) maps. Based on the acquired standard sagittal anatomical T2 TSE-weighted images and the MMRE scan, IVDs were manually segmented on the SWS maps and evaluated with the Pfirrmann grading scale of disc degeneration (1–5) performed by an experienced (+10 years) MRI spine researcher. Spearman's rank correlation was calculated to investigate the correlation between stiffness (SWS) and DDD. Statistical significance was assumed when the *p* value was < 0.01.

**Figure 2:**
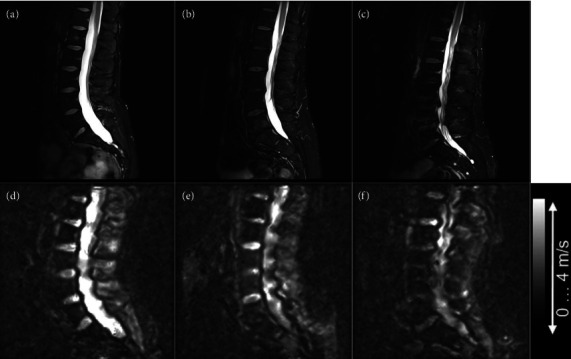
Sagittal dixon images (a–c) and SWS maps (d–f) from three male participants: 32 (a, d), 50 (b, e), and 60 (c, f) years of age demonstrate decreased stiffness with progressive DDD.

**Figure 3:**
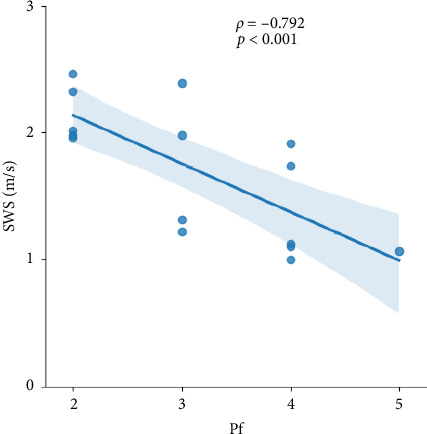
Relationship between Pfirrmann degeneration scores and tissue stiffness of the IVD (SWS) showing a significant negative correlation.

Figure 2 displays sagittal T2 Dixon images and SWS maps—a surrogate parameter of tissue stiffness—illustrating the feasibility of high-resolution in vivo MMRE across all five lumbar discs. A plot of SWS against Pfirrmann grades (Figure 3, Table 1) demonstrates an inverse relationship between increasing IVD stiffness and progressive DDD (Spearman's rank correlation coefficient *ρ* = −0.792, *p* < 0.001).

## 3. Discussion

To our knowledge, this is the first case report describing MMRE results from the spine using a dual-actuator setup combined with the k-MDEV inversion algorithm. The versatility and effectiveness using MMRE in assessing tissue stiffness and mechanical properties have been demonstrated across different body parts, including the abdomen, pelvic organs, and brain [13, 17, 18]. Our results suggest the feasibility of the MMRE system, including its custom-designed smaller dual-actuators, in generating sufficiently strong shear wave magnitudes to produce high-resolution elastography results over multiple frequencies in deep spinal tissues. Additionally, we show that spine MMRE is sensitive to the progression of DDD. Due to the close relationship between the mechanical properties of the IVD and its function, MMRE has the potential to be more sensitive in detecting structural changes than conventional MRI methods. Our data is congruent with a prior MMRE study [10] and mechanical IVD testing [19] that demonstrated an inverse relation between the severity of DDD and stiffness. Compared to the MMRE study [10], our elastograms demonstrated superior visual image quality that we attribute to our setup's dual-actuator design, magnitude of displacement and superior inversion algorithm. While k-MDEV shares some common noise-handling approaches with other MRE inversion algorithms in the initial smoothing process, its distinguishing features lie in the later stages of processing. These include: (i) the use of a multifrequency approach that combines data from multiple mechanical excitation frequencies, (ii) the implementation of a wavenumber-based algorithm for calculating the complex shear modulus, and (iii) the application of a dual-parameter fit to separate the real and imaginary parts of the complex shear modulus, allowing for simultaneous calculation of elasticity and viscosity. More details regarding the algorithm's supremacy within low-SNR regions due to its noise-robust wave number recovery strategy are given in a previous paper [16]. In contrast, recent findings using a single-frequency MRE setup have shown a direct relation between increasing degeneration and increasing stiffness [20]. However, an acknowledged limitation of this single-frequency MRE study was the use of a principal frequency analysis method, which did not allow for the creation of stiffness maps accounting for spatial variations in tissue stiffness. Although these contradictory results have yet to be resolved, ongoing discussions suggest a potentially significant effect of MRE postprocessing approaches. Although our study has shown promising results in evaluating in vivo IVD properties, there are limitations. The accuracy of mechanical property measurements is significantly influenced by the boundary and preload conditions. This factor also significantly impacts in vivo MMRE measurements, making our findings dependent on the selected frequency range and wave SNR. To further validate the consistency of our IVD mechanical parameters, additional data collection is necessary.

## 4. Conclusions

MMRE postprocessed with k-MDEV inversion allows for the measurement of in vivo mechanical properties of IVDs and may provide additional information in disc degeneration assessment beyond standard morphological changes. Prior to the clinical use of this technique, further studies should be conducted to evaluate the reproducibility and repeatability of spinal MMRE and its potential confounders.

## Figures and Tables

**Table 1:** MMRE-derived surrogate parameter for tissue stiffness (SWS) and Pfirrmann score for all segmented IVD levels.

Participant	IVD level	SWS in m/s	Pfirrmann score
Male, 60 years	L1/L2	1.336	3
	L2/L3	1.09	4
	L3/L4	1.052	5
	L4/L5	1.117	4
	L5/S1	1.193	3

Male, 32 years	L1/L2	2.426	2
	L2/L3	2.314	2
	L3/L4	2.003	2
	L4/L5	1.972	2
	L5/S1	2.053	2

Male, 50 years	L1/L2	1.738	4
	L2/L3	2.4	3
	L3/L4	1.895	4
	L4/L5	1.998	3
	L5/S1	0.993	4

## Data Availability

Deidentified data used to support the findings of this study are available from the corresponding author upon reasonable request.
